# Tumor cell-released autophagosomes (TRAPs) promote immunosuppression through induction of M2-like macrophages with increased expression of PD-L1

**DOI:** 10.1186/s40425-018-0452-5

**Published:** 2018-12-18

**Authors:** Zhi-Fa Wen, Hongxiang Liu, Rong Gao, Meng Zhou, Jie Ma, Yue Zhang, Jinjin Zhao, Yongqiang Chen, Tianyu Zhang, Fang Huang, Ning Pan, Jinping Zhang, Bernard A. Fox, Hong-Ming Hu, Li-Xin Wang

**Affiliations:** 1 Department of Microbiology and Immunology, Medical School of Southeast University, 87 Dingjiaqiao Rd, Nanjing, 210009 People’s Republic of China; 2 Institutes of Biology and Medical Sciences, Soochow University, Suzhou, 215123 People’s Republic of China; 3 Robert W. Franz Cancer Research Center, Earle A. Chiles Research Institute, Providence Portland Medical Center, 2N81 North Pavilion, 4805 N.E. Glisan St, Portland, OR 97213 USA

**Keywords:** Tumor cell-released autophagosomes (TRAPs), TAMs, PD-L1, IL-10, MyD88, T cell, Tumor microenvironment

## Abstract

**Background:**

Tumor-associated macrophages (TAMs) facilitate tumor progression via establishment of an immunosuppressive tumor microenvironment (TME). However, it is poorly understood how tumor cells could functionally modulate TAMs. Our previous work indicated that tumor cell-released autophagosomes (TRAPs), a type of LC3-II^+^ double-membrane extracellular vesicles (EVs) was sufficient to suppress anti-tumor immune responses by inducing IL-10-producing B cells and immune suppressive neutrophils. Here, we hypothesized that TRAPs may participate in regulating macrophage polarization.

**Methods:**

TRAPs isolated from multiple murine tumor cell lines and pleural effusions or ascites of cancer patients were incubated with bone marrow-derived macrophages (BMDMs) and monocytes, respectively. Cellular phenotypes were examined by flow cytometry, ELISA and quantitative PCR. TRAPs treated BMDMs were tested for the ability to suppress T-cell proliferation in vitro, and for promotion of tumor growth in vivo. Transwell chamber and neutralization antibodies were added to ascertain the inhibitory molecules expressed on BMDMs exposed to TRAPs. Knockout mice were used to identify the receptors responsible for TRAPs-induced BMDMs polarization and the signaling mechanism was examined by western blot. Autophagy-deficient tumors were profiled for phenotypic changes of TAMs and IFN-γ secretion of T cells by flow cytometry. The phenotype of monocytes from pleural effusions or ascites of cancer patients was assessed by flow cytometry.

**Results:**

TRAPs converted macrophages into an immunosuppressive M2-like phenotype characterized by the expression of PD-L1 and IL-10. These macrophages inhibited the proliferation of both CD4^+^ and CD8^+^ T cells in vitro, and promoted tumor growth mainly through PD-L1 in vivo. TRAPs-induced macrophage polarization was dependent on TLR4-mediated MyD88-p38-STAT3 signaling. In vivo studies indicated that disruption of autophagosome formation in B16F10 cells by silencing the autophagy gene *Beclin1* resulted in a remarkable delay in tumor growth, which was associated with reduced autophagosome secretion, TAMs reprogramming and enhanced T cell activation. Moreover, the levels of LC3B^+^ EVs appeared to correlate significantly with up-regulation of PD-L1 and IL-10 in matched monocytes from effusions or ascites of cancer patients, and TRAPs isolated from these samples could also polarize monocytes to an M2-like phenotype with increased expression of PD-L1, CD163 and IL-10, decreased expression of HLA-DR, and T cell-suppressive function.

**Conclusions:**

These findings suggest the TRAPs-PD-L1 axis as a major driver of immunosuppression in the TME by eliciting macrophage polarization towards an M2-like phenotype, and highlight the potential novel therapeutic approach of simultaneously targeting autophagy and PD-L1.

**Electronic supplementary material:**

The online version of this article (10.1186/s40425-018-0452-5) contains supplementary material, which is available to authorized users.

## Background

Tumor-associated macrophages (TAMs) represent a major constituent of the leukocytes infiltrate in the tumor microenvironment (TME) where they mostly display tumor-promoting functions by facilitating tumor proliferation and survival, angiogenesis, metastasis, as well as immune suppression [1]. High infiltration of TAMs generally predicts unfavorable prognosis for most human tumors [2–8], with the exception of colorectal cancer [9]. TAMs are phenotypically heterogeneous and functionally diverse and form a continuous spectrum of polarization states, unlike the oversimplified M1/M2 classification [10]. During tumor progression, TAMs are polarized from a M1-like anti-tumor to a M2-like pro-tumor phenotype [11], highlighting that reprogramming TAMs could be employed as a rational cancer therapeutic strategy. TAMs suppress immune responses through multiple mechanisms. For instance, TAMs are capable of producing immunosuppressive mediators/cytokines (arginase and IL-10) [12, 13] and expressing inhibitory molecules (PD-L1 and PD-L2) [14, 15]. Tumor-derived soluble mediators such as chemokines (CCL2, CCL5, CXCL12), colony-stimulating factor-1 (CSF-1), TGF-β, IL-10, prostaglandin E2 (PGE2) and metabolites (lactate) likely contribute to the development of TAMs with M2-like phenotype and tumor-promoting properties [16]. Recent studies indicated that, in addition to soluble factors, tumor-derived exosomes could also modulate macrophage cytokine profile and phenotype [17–21]. However, little is known about how TRAPs affect macrophage polarization and function in vitro and in vivo.

Autophagy is an evolutionarily conserved and tightly orchestrated intracellular catabolic process in which misfolded proteins or damaged organelles are sequestered in autophagosomes and ultimately fuse to lysosomes for degradation and recycling [22, 23]. In contrast to degradative autophagy, secretory autophagy could bypass lysosome fusion and exports a wide range of cytoplasmic substrates, such as IL-1β, HMGB1, amyloid beta, microorganism and autophagic vacuoles [24–26]. A recent study showed that, during bacterial infection, lysozyme-containing autophagosomes undergo fusion with the apical surface of Paneth cells and secretion into the intestinal lumen [27]. Autophagy is normally executed at a basal level, but is strongly enhanced in established tumors under hypoxic stress and nutrient deprivation [28]. Although the role of autophagy in cancer is controversial, it is generally accepted that autophagy prevents cancer development in pre-malignant lesions, but promotes advanced cancer growth [29]. Indeed, a higher autophagic flux is associated with poor response to chemotherapy and worse overall survival in melanoma patients [30]. Whereas the functional consequence of autophagy inhibition in tumor cells has been established, the outcome of targeting autophagy in the TME, especially in TAMs, is not fully defined.

Our previous studies have verified the effects of TRAPs on the immunological functions of B cells and neutrophils. TRAPs can be readily taken up by B cells, subsequently induce IL-10 production, which potentially suppresses T-cell proliferation and antitumor responses [31]. Treatment of human neutrophils with TRAPs promoted the generation of reactive oxygen species (ROS) through macropinocytosis, contributing to the inhibition of T-cell activation and proliferation in a ROS-dependent manner [32]. In this study, we demonstrate that TRAPs are sufficient to induce an M2-like phenotype in macrophages characterized by increased expression of PD-L1 and IL-10 via a mechanism involving TLR4-MyD88-p38-STAT3 signaling. TRAPs-induced macrophages are highly efficient at inhibiting T cell proliferation and promoting tumor growth mainly through PD-L1. The immunomodulatory effects of cancer patients-derived TRAPs on human monocytes are also confirmed. In addition, the tumor-supporting properties of endogenous TRAPs were further demonstrated in vivo in autophagy-defective tumor models. Overall, these findings uncover a crucial role of TRAPs on induction of immunosuppressive TAMs.

## Methods

### Mice, cell lines and reagents

Wild-type (WT) C57BL/6 and BALB/c mice were purchased from the Comparative Medicine Center of Yangzhou University (Yangzhou, China). TLR2 knockout (KO), TLR4 KO, MyD88 KO and OT-I mice were purchased from the Nanjing Biomedical Research Institute of Nanjing University (Nanjing, China). PD-L1 KO mice were purchased from The Jackson Laboratory (MMRRC stock # 32234, Bar Harbor, USA). All animal experiments were approved by the Animal Care and Use Committee of Southeast University.

4T1, B16F10, EL4 and Hepa 1–6 were cultured in RPMI 1640 (HyClone) supplemented with 10% FBS (Gibco), 0.05 mM 2-ME (Gibco) and 50 μg/ml Gentamicin (Lonza). Raw264.7 were cultured in complete DMEM (HyClone) medium. B16F10 tumor cells stably transduced with Beclin1-specific short hairpin RNA (shRNA) (BECN1-KD B16F10) and scrambled shRNA (Ctrl-B16F10) were generated previously. All cell lines were detected for Mycoplasma every 2 weeks and were negative prior to use.

LPS, Chloroquine diphosphate, Chlorophenol red-β-D-galactopyranoside (CPRG), and Proteinase K were purchased from Sigma (St. Louis, USA). SB203580 and Stattic were purchased from MCE (Shanghai, China). Recombinant Murine M-CSF, IFN-γ and IL-4 were purchased from PeproTech (Rocky Hill, USA). CFSE was purchased from Invitrogen.

### Patients

This study was approved by the Ethics Committee for Human Studies of Southeast University and performed under protocol 2016ZDKYSB112. Peripheral blood and malignant pleural effusions and ascites were collected from patients with pathologically diagnosed with multiple cancer types. The clinical pathological characteristics of enrolled patients are presented in (Additional file 1: Table S1).

### TRAPs isolation and characterization

TRAPs were prepared from tumor cells as previously described [33]. Briefly, cells culture supernatant was centrifuged at 450 g for 7 min to get rid of dead cells and debris. The supernatant was further centrifuged at 12000 g for 15 min to pellet TRAPs-containing large extracellular vesicles (EVs). Subsequently, EVs were washed twice with PBS consisting of 20 mM NH_4_Cl plus 2.5 mM EDTA and TRAPs were isolated from EVs. TRAPs were finally resuspended in PBS and total protein concentration was measured using a BCA Protein Assay Kit (Pierce) following the manufacturer’s instructions. For isolation of TRAPs from B16F10 cells transfected with GFP-LC3, GFP-LC3-marked autophagosomes were pulled down by using sheep anti-mouse IgG Dynabeads (Invitrogen) in combination with mouse anti-GFP antibody (Abcam) according to the manufacturer’s recommendations. For isolation of TRAPs from other tumor cell lines and malignant pleural effusions and ascites of cancer patients, autophagosomes were pulled down by using anti-biotin MicroBeads (Miltenyi) in combination with biotinylated LC3B Rabbit mAb (Cell Signaling Technology). For flow cytometry identification, TRAPs were stained with rabbit anti-LC3B antibody (Sigma), followed by R-PE conjugated anti-Rabbit IgG secondary antibody (proteintech), or stained with PE-LC3B mAb (Cell Signaling Technology). The purity and LC3-II content were verified by flow cytometry and Western blot, respectively.

### Transmission Electron microscopy

TRAPs samples for transmission electron microscopy were prepared as previously described [31]. Briefly, the samples were fixed in 2.5% glutaraldehyde, 100 mM sodium cacodylate, 0.064% picric acid, 0.1% ruthenium red and 1.6% paraformaldehyde, then post-fixed with a mixture of osmium tetroxide plus potassium ferricyanide and finally embedded in Epon. A series of TRAPs images were taken with a JEM-1011 (JEOL, Tokyo, Japan) transmission electron microscope at 60 kV.

### Primary macrophage isolation

Bone marrow-derived-macrophages (BMDMs) and Peritoneal macrophages were generated according to previous literature [34]. Briefly, Bone marrow cells from the femurs and tibias were cultured in RPMI 1640 supplemented with 10% FBS, 50 μg/ml Gentamicin and 20 ng/ml M-CSF. BM cells were adjusted to a concentration of ~ 1 × 10^6^/ml and were added into 100 mm petri dishes. On day 3, culture medium was discarded and the attached cells were washed once with cold PBS, fresh complete medium containing M-CSF was then added. BMDMs were harvested on day 7 and used for subsequent experiments. For peritoneal macrophages, 6 ml cold complete medium was injected into peritoneal cavity and macrophages were harvested by peritoneal lavage. The cells were centrifuged at 400 g for 10 min and seeded in 24-well plates. Macrophages were allowed to adhere for 2 h at 37 °C and the suspension cells were washed extensively with warm medium prior to use.

### ELISA

The concentration of IL-6, IL-1β, IL-12 p70 and IL-10 produced by mouse macrophages was detected by IL-6, IL-1β, IL-12 p70 and IL-10 ELISA kits (eBioscience), respectively, in accordance with manufacturer’s guidelines. Human IL-10 and IFN-γ were measured by ELISA kits (eBioscience).

### Real-time quantitative PCR

Total RNA was extracted from macrophages using an RNAprep pure Cell / Bacteria Kit (TIANGEN), and was subsequently reverse transcribed into cDNA with PrimeScript RT reagent Kit (Takara). qRT-PCR was then conducted on the StepOnePlus Real-Time PCR System (Applied Biosystems, Foster City, USA) with TB Green Premix Ex Taq II (Takara). Primers were synthesised by GenScript (Nanjing, China) and the sequences for each gene were presented in (Additional file 1: Table S2). Relative gene expression was calculated using the 2^-ΔΔCt^ method normalizing to GAPDH expression.

### Western blot

The cells were lysed in RIPA lysis buffer (Millipore) containing protease (Bimake) and phosphatase (Bimake) inhibitors. The samples were resolved by SDS-PAGE and transferred to PVDF membrane after blocking, the membranes were incubated with primary antibodies overnight at 4 °C and then exposed to secondary antibodies. Protein bands were visualized using West Femto Substrate Trial Kit (Thermo Scientific). The primary antibodies included anti-p38 MAPK (Cell Signaling Technology), anti-phospho-p38 MAPK (Thr180/Tyr182) (Cell Signaling Technology), anti-STAT3 (Abcam), anti-phospho-STAT3 (Tyr705) (Abcam), anti-LC3B (Sigma), anti-Beclin1 (proteintech), anti-β-Tubulin (Cell Signaling Technology), and anti-GAPDH (proteintech). The secondary antibodies were goat anti-mouse IgG HRP (proteintech) and goat anti-rabbit IgG HRP (proteintech).

### Flow cytometry and antibodies

Single-cell suspensions were blocked with mouse FcR blocking reagent (Miltenyi Biotec) for 10 min at 4 °C prior to surface staining. Cell viability was assessed by Fixable viability dye eFluor 520 (eBioscience) to exclude dead cells. The following anti-mouse antibodies were used: FITC-CD11b, PE-F4/80, APC-F4/80, FITC-CD45, PerCP-eFluor 710-MHC Class II (I-A/I-E), PerCP-eFluor 710-CD3, FITC-CD3e, FITC-CD4, APC-CD4, APC-CD8a, PE-PD-L2, PE-B7-H2, PE-B7-H3, PE-B7-H4, PE-TIM-4, PE-VISTA from eBioscience; APC-CD206, PE-PD-L1, APC-CD86, PE/Cy7 Ki-67 from Biolegend; V450-CD4, BV510-CD8, PE-IFN-γ from BD. The following anti-human antibodies were used: PerCP-eFluor 710-CD3, APC-CD4, APC-CD8a from eBioscience; FITC-CD14, PE-PD-L1, APC-CD163, APC-CD86, FITC-HLA-DR, PE-Cy7-CD163, PE-CD25, PE-IFN-γ from Biolegend; FITC-CD8, PE-IL-10 from BD. For intracellular staining, cells were fixed and permeabilized with the Fixation/Permeabilization solution kit (BD). All flow cytometry data was acquired on FACSCalibur or LSRFortessa (BD, San Jose, USA) and analyzed by FlowJo V10 (TreeStar, Ashland, USA).

### In vitro T cell proliferation and activation assay

Mouse T cells were isolated from spleens using the Mouse Pan T Kit (Invitrogen), labeled with carboxyfluorescein succinimidyl ester (CFSE) (2 μM; Invitrogen), then were plated in 48-well anti-CD3 (5 μg/ml; BD) coated plates plus soluble anti-CD28 (1 μg/ml; BD) added to the medium. According to protocol described previously [35], BMDMs pretreated with TRAPs were added 3 h after T cell activation at indicated ratios. Cells were cocultured with or without neutralizing monoclonal antibodies against PD-L1, IL-10 or rat IgG isotype control (eBioscience). For transwell assay, TRAP-pretreated macrophages and T cells were seeded in the top chamber and bottom chamber of the transwell insert, respectively. For antigen-specific T cell proliferation, TRAP-pretreated macrophages were cocultured with CFSE labeled OT-I splenocytes in the presence of OVA_257–264_ peptide (1 μg/ml; GL Biochem). 72 h later, CD4^+^ and CD8^+^ T cell division was determined by flow cytometry. For human T cell proliferation assay, peripheral blood mononuclear cells (PBMCs) were isolated from healthy donors by Ficoll-Paque PLUS (GE Healthcare), and CD14^+^ monocytes and autologous CD3^+^ T cells were purified from PBMCs using the Human monocyte isolation kit (Stemcell) and Human CD3 Kit (Invitrogen), respectively. Subsequently, monocytes were exposed to TRAPs derived from cancer patients for 72 h, and then cocultured with CFSE labeled CD3^+^ T cells at a ratio of 1:3 in 96-well anti-CD3 (5 μg/ml; BD) coated plates plus soluble anti-CD28 (1 μg/ml; BD) for 5 d. For T cell activation assay, TRAP-pretreated monocytes were incubated with autologous T cells at a ratio of 1:3 in anti-CD3 (0.5 μg/ml; BD) coated plates for 20 h, CD25 expression of CD4^+^ and CD8^+^ T cells was examined by flow cytometry. Breferdin A plus monensin was added during the last 8 h of culture, the frequency of IFN-γ^+^ T cells was determined by intracellular staining.

### In vivo tumor models

For melanoma models, 6–8-week-old female C57BL/6 mice were implanted subcutaneously into the right flank with 5 × 10^5^ BECN1-KD B16F10 cells or Ctrl-B16F10 cells. Tumor growth was measured using a caliper every third day, and mice were euthanized when the area of tumors reached 150 mm^2^. For co-injection experiments, B16F10 cells (5 × 10^5^) were mixed with 2.5 × 10^5^ WT or PD-L1 KO BMDMs treated with or without TRAPs from B16F10 cells, and then co-injected subcutaneously into mice. To analyze tumor immune response, mice were sacrificed 15–20 d after tumor cell inoculation. Draining lymph nodes (dLNs) or spleens were processed through a 70 μm cell strainer with syringe plungers. Tumors were excised, minced, and enzymatically dissociated in DMEM containing Collagenase IV (1 mg/ml; Sigma) and DNase I (20 U/ml; Sigma) in a shaking incubator at 37 °C for 30 min. Cell suspensions were subsequently passed through a cell strainer. For ex vivo functional assays, tumor samples were stimulated with Cell Stimulation Cocktail (eBioscience) for 5 h.

### Statistical analysis

Data are expressed as Mean ± SEM. Statistically significance of differences was analyzed using GraphPad Prism 7 (GraphPad Software, La Jolla, USA) by unpaired Student’s *t* test, one-way ANOVA or two-way ANOVA. Correlation coefficients and their significance were calculated by two-tailed Spearman’s rank correlation. A *P* value of < 0.05 is considered statistically significant.

## Results

### TRAPs polarize macrophages to M2-like phenotype in vitro and in vivo

**Fig. 1:**
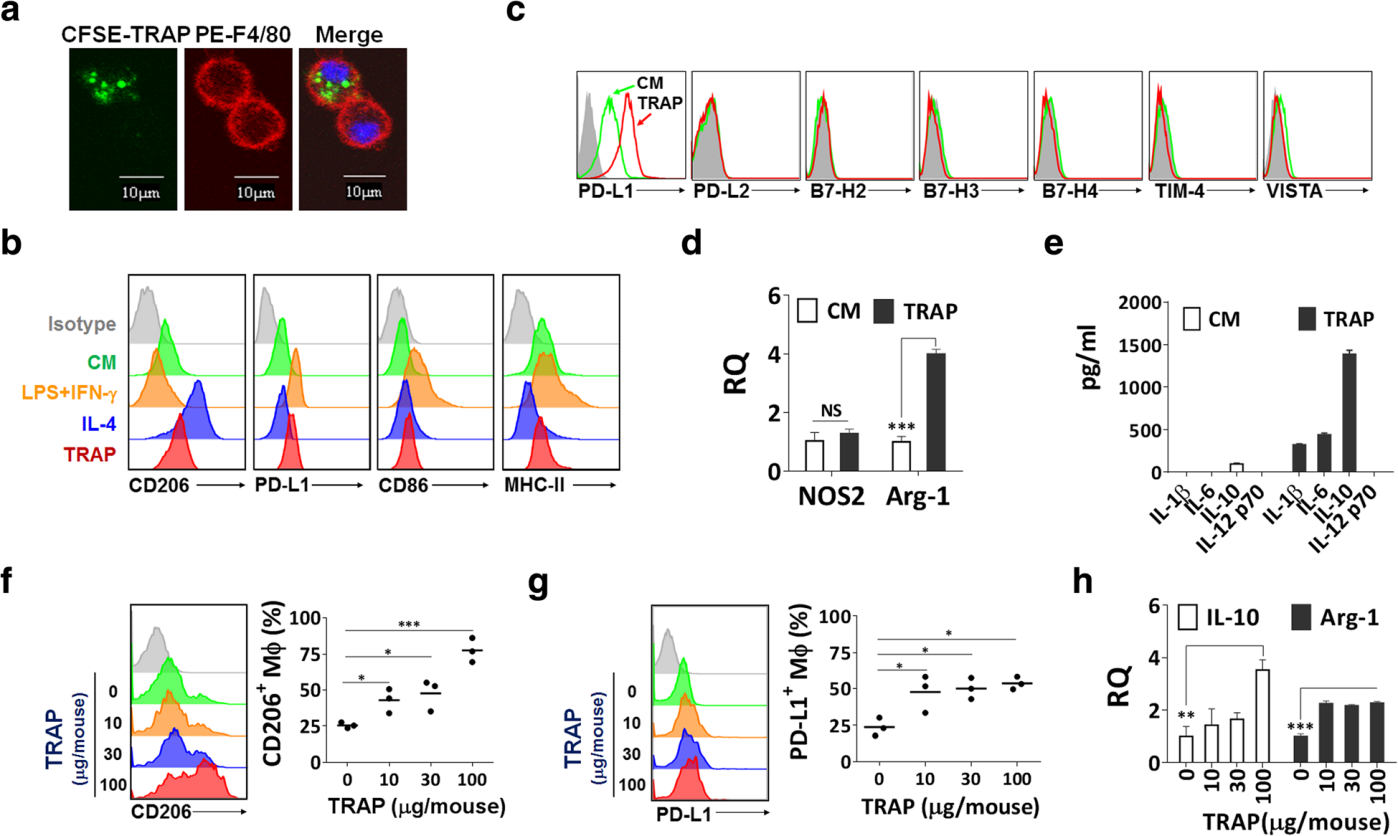
TRAPs polarize macrophages toward an M2-like phenotype in vitro and in vivo. **a** Confocal images of BMDMs treated with CFSE-labeled TRAPs (green). After incubation with TRAPs (10 μg/ml) for 0.5 h, BMDMs were stained with PE-F4/80 antibody (red) and DAPI (blue). Scale bar, 10 μm. **b** Expression analysis of CD206, PD-L1, CD86 and MHC II by flow cytometry. BMDMs were stimulated with LPS (100 ng/ml) + IFN-γ (20 ng/ml), IL-4 (20 ng/ml) or TRAPs (10 μg/ml) for 48, 48 or 72 h, respectively. **c** Flow cytometry analysis of PD-L2, B7-H2, B7-H3, B7-H4, Tim-4 and VISTA for BMDMs after incubating with TRAPs for 48 h. **d** Expression analysis of *NOS2* and *Arg1* mRNA in BMDMs treated with TRAPs (10 μg/ml) for 6 h by qRT-PCR. **e** ELISA detection of IL-1β, IL-6, IL-10 and IL-12p70 produced by BMDMs exposed to TRAPs (10 μg/ml) for 72 h. **f-h** Mice (*n* = 3) were intraperitoneally injected with four different doses of TRAPs (0, 10, 30 and 100 μg) at day 0 and the phenotype of peritoneal macrophages was analyzed at day 3. Expression of CD206 (**f**) and PD-L1 (**g**) on macrophages (F4/80^+^CD11b^+^) was determined by flow cytometry. **h** mRNA expression level of *IL-10* and *Arg1* in purified macrophages was detected by qRT-PCR. Results are representative of three independent experiments. *p < 0.05, **p < 0.01 and ***p < 0.001 by unpaired *t* test (**d**, **f**, **g** and **h**)

Similar to the characteristics of autophagosomes [22], TRAPs from culture supernatant of the murine melanoma cell line B16F10 were found to possess a double membrane structure with diameters ranging from 300 to 900 nm and express LC3-II (Additional file 2: Figure S1a-c). To examine the interaction between TRAPs and macrophages, TRAPs labeled with the green fluorescent dye CFSE were incubated with bone-marrow-derived macrophages. TRAPs uptake was observed as early as 30 min and increased thereafter by confocal microscopy analysis (Fig. 1a).

In order to determine the phenotype of macrophages after TRAPs treatment, flow cytometric analysis was conducted to detect the expression of M1 (CD86, MHC-II) and M2 (CD206) markers. TRAPs substantially increased CD206 and slightly reduced MHC-II expression, but failed to induce CD86 (Fig. 1b). TRAPs also upregulated the expression of PD-L1 but not other co-inhibitory ligands [10], including PD-L2, B7-H2, B7-H3, B7-H4, TIM-4, and VISTA, on macrophages (Fig. 1c). Furthermore, RT-PCR analysis showed that the representative M2 gene arginase-1 (*Arg1)*, but not the M1 gene nitric oxide synthase 2 (*NOS2*), was increased (Fig. 1d). In addition, TRAPs-treated macrophages secreted very high level of IL-10, low levels of IL-1β and IL-6, and no IL-12p70 (Fig. 1e). TRAPs also acted in a dose-dependent manner to induce the expression of PD-L1 and IL-10 (Additional file 2: Figure S2a and b).

In addition to bone-marrow-derived macrophages, the macrophage cell line RAW264.7 cells as well as peritoneal macrophages showed increased PD-L1 expression and IL-10 production after TRAPs treatment (Additional file 2: Figure S2c and d). TRAPs-induced PD-L1 and IL-10 expression did not appear to be an isolated phenomenon, as TRAPs from a variety of murine tumor cell lines had similar effects on macrophages (Additional file 2: Figure S2e and f).

To further examine whether TRAPs regulate macrophages polarization in vivo, TRAPs isolated from B16F10 tumor cells were intraperitoneally injected into C57BL/6 mice. Mice that received 10 μg TRAPs had increased expression of CD206 and PD-L1, and elevated *Il10* and *Arg1* transcripts in peritoneal macrophages (Fig. 1f–h), consistent with our in vitro observations suggesting that TRAPs are M2-like macrophage polarizers.

### TRAPs-educated macrophages exert immunosuppressive functions in vitro

**Fig. 2:**
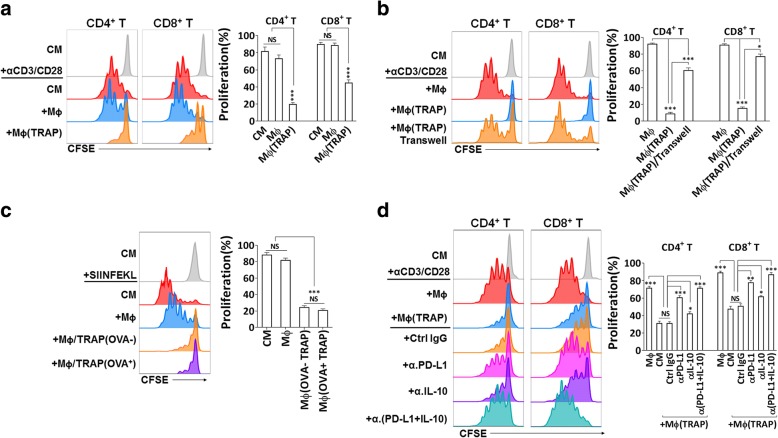
TRAPs exposed macrophages inhibit T cell proliferation via PD-L1 and IL-10. **a-d** Representative determination of T cell proliferation by flow cytometry. **a**, **b**, **d** CFSE-labeled purified CD3^+^ T cells were activated by immobilized anti-CD3 plus soluble anti-CD28 mAb, and were either cultured alone or cocultured with BMDMs pretreated with or without TRAPs at a ratio of 5:1 for 3 d. **b** A transwell chamber was used to separate BMDMs from T cells. **d** BMDMs were cocultured with T cells in the presence of anti-PD-L1 mAb, anti-IL-10 mAb or control IgG. **c** BMDMs were left untreated or pretreated with 10 μg/ml B16F10 TRAPs or B16F10-OVA TRAPs for 2 d, and loaded with OVA_257–264_ peptide SIINFEKL (1 μg/ml) for 2 h, washed, were then incubated with CFSE-labeled OT-I splenocytes for 3 d at a ratio of 1:20. Data are representative of three independent experiments. *p < 0.05, **p < 0.01, and ***p < 0.001 by unpaired *t* test (**a**-**d**)

To ascertain the functional activities of TRAPs-induced macrophages, CFSE-labeled naive T cells were cocultured with varying numbers of macrophages pretreated with or without TRAPs in the presence of anti-CD3/anti-CD28. Notably, TRAPs-treated macrophages, but not control macrophages, inhibited CD4^+^ and CD8^+^ T cell proliferation (Fig. 2a and Additional file 2: Figure S3a). Suppression was partially dependent upon cell contact, as TRAPs-exposed macrophages had reduced suppressive activity on CD4^+^ and CD8^+^ T-cell proliferation when separated from T cells by transwell (Fig. 2b).

Macrophages treated with either OVA^+^-TRAPs or OVA^−^-TRAPs were equally capable of abrogating OT-I T cell proliferation induced by the cognate OVA_257–264_ peptide, indicating that TRAPs-stimulated macrophages could also inhibit antigen-specific CD8^+^ T cell proliferation (Fig. 2c). PD-L1 blockade, and to a lesser extent, IL-10 neutralization, significantly restored T cell proliferation. More importantly, dual PD-L1/IL-10 blockade completely abrogated the suppressive function of TRAPs-treated macrophages on T cell proliferation (Fig. 2d).

Macrophages exposed to EL4 cell-derived TRAPs were pulsed with OVA_257–264_ peptide and subsequently co-cultured with the OVA_257–264_-specific B3Z hybridoma cells which constitutively express a high level of PD-1. TRAPs-treated macrophages were less efficient in activating B3Z cells than control macrophages. IL-10 blockade partially restored B3Z activation, and PD-L1 blockade even augmented the B3Z activation (Additional file 2: Figure S3b). Taking together, the mechanism for TRAPs-treated macrophages appeared to predominately involve PD-L1/PD-1 signaling, with IL-10 playing a minor role.

### The immunosuppressive function of TRAPs-educated macrophages is TLR4-MyD88-dependent

**Fig. 3:**
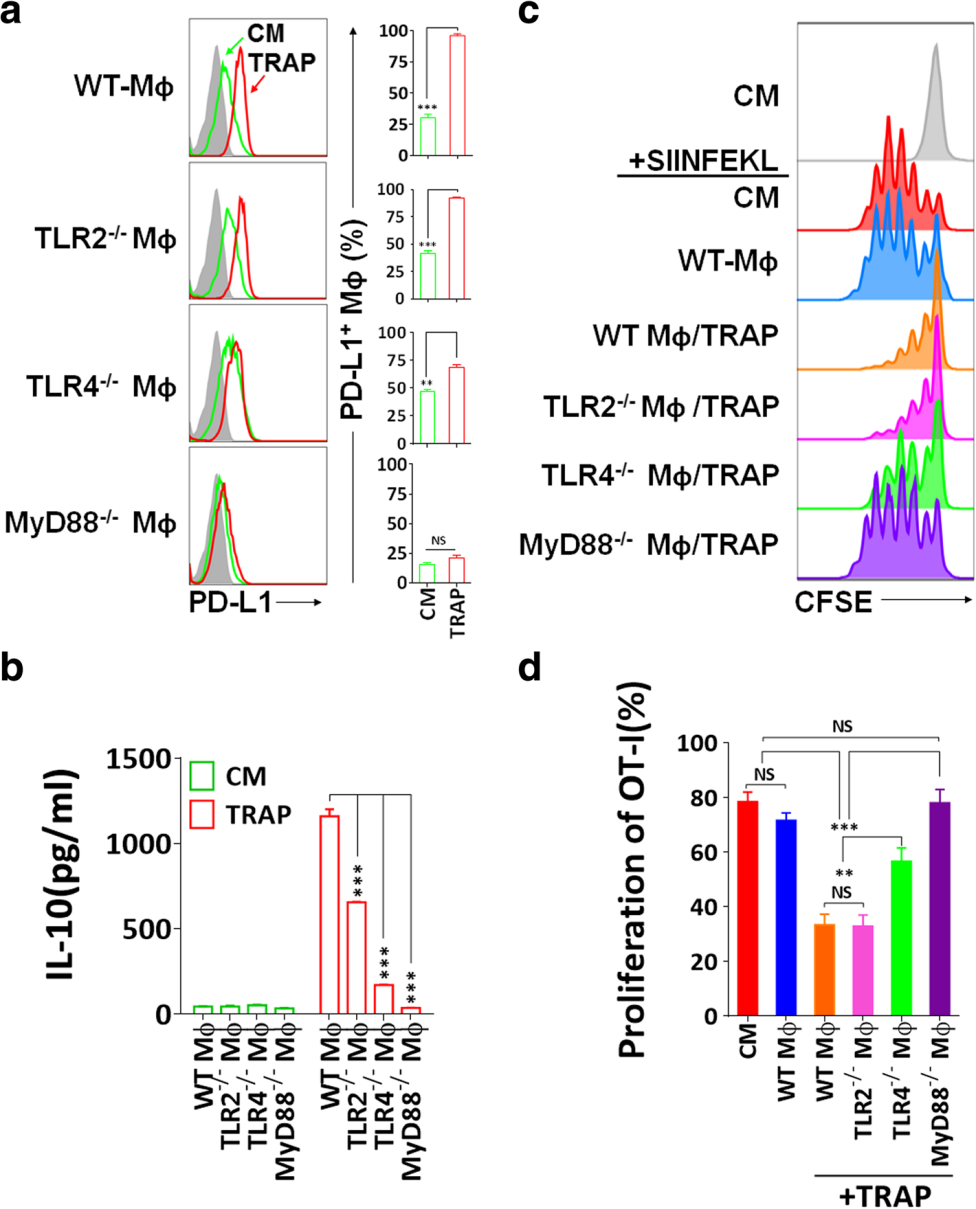
Immunosuppression mediated by TRAPs treated BMDMs is dependent on TLR4-MyD88 signaling. **a** Expression analysis of PD-L1 by flow cytometry. BMDMs derived from WT, TLR2^−/−^, TLR4^−/−^, or MyD88^−/−^ mice were incubated with TRAPs (10 μg/ml) for 48 h. **b** ELISA detection of IL-10 secreted from BMDMs exposed to TRAPs (10 μg/ml) for 72 h. **c**, **d** Detection of OT-I CD8^+^ T cell division by flow cytometry. BMDMs derived from WT, TLR2^−/−^, TLR4^−/−^, or MyD88^−/−^ mice were left untreated or pretreated with B16F10 TRAPs (10 μg/ml) for 2 d, and pulsed with peptide SIINFEKL (1 μg/ml) for 2 h, washed, and followed by coculture with CFSE-labeled OT-I splenocytes for 3 d at a ratio of 1:20. Data (mean ± SEM) are representative of three independent experiments. **p < 0.01, ***p < 0.001 by unpaired *t* test. (**b** and **d**)

TRAPs are enriched of a broad array of endogenous damage-associated molecular pattern molecules (DAMPs), which may trigger innate immune responses through TLRs [31]. Moreover, TLR ligation of antigen-presenting cells (APCs) was critical for the induction of PD-L1 and IL-10 [36]. To determine the mechanism of TRAPs-mediated macrophage polarization, BMDMs from mice deficient in TLR2, TLR4, or MyD88 were incubated with TRAPs. TRAPs-induced PD-L1 expression was completely MyD88-dependent, and PD-L1 upregulation was markedly diminished due to TLR4, but not TLR2 deficiency (Fig. 3a). IL-10 secretion was impaired in *Tlr4*^−/−^ or *Myd88*^−/−^ macrophages, although reduced IL-10 release was also observed in *Tlr2*^−/−^ macrophages (Fig. 3b).

In addition, *Tlr4*^−/−^ or *Myd88*^−/−^, but not *Tlr2*^−/−^ macrophages treated with TRAPs had diminished capability of inhibiting OT-I proliferation (Fig. 3c and d). Collectively, these results indicate that TLR4-MyD88, but not TLR2, underlies the suppressive function of TRAPs-educated macrophages on T cell proliferation.

### p38-STAT3 signaling promotes TRAPs-mediated macrophage polarization

**Fig. 4:**
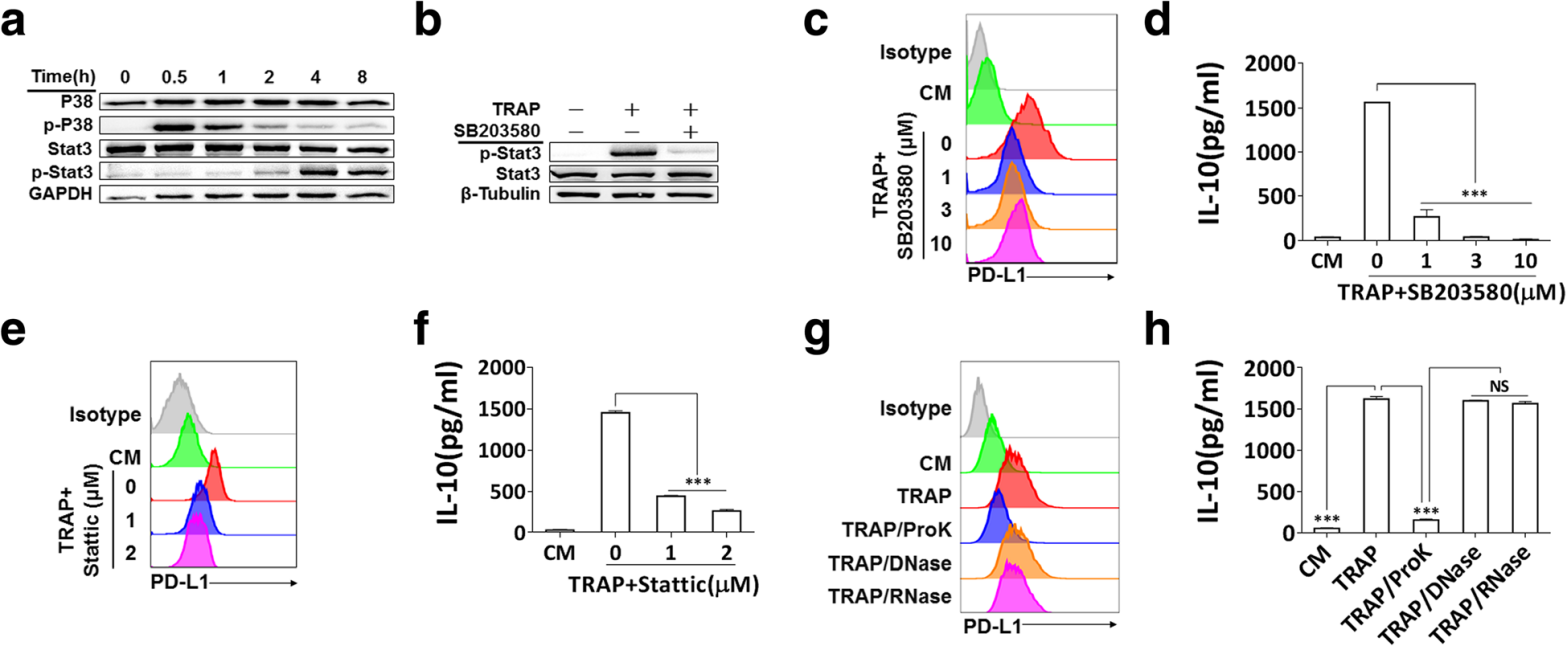
p38-STAT3 signaling in BMDMs and protein fraction in TRAPs are essential for induction of PD-L1 and IL-10. **a** BMDMs were exposed to TRAPs (10 μg/ml) at indicated time points. Cell lysates were analyzed for p38, p-p38, STAT3 and p-STAT3 by western blot. GAPDH was used as a loading control. **b** BMDMs were pretreated with p38 inhibitor SB203580 (3 μM) for 1 h, and then co-incubated with TRAPs (10 μg/ml) for 4 h. Expression of STAT3 and p-STAT3 was detected by western blot. **c** BMDMs were exposed to SB203580 at described concentrations for 1 h, and followed by incubation with TRAPs (10 μg/ml) for 72 h. PD-L1 expression was determined by flow cytometry, and (**d**) the production of IL-10 was assessed by ELISA. **e** BMDMs were pretreated with STAT3 inhibitor Stattic (1 and 3 μM) for 1 h, and then stimulated with TRAPs (10 μg/ml) for 72 h. PD-L1 expression and (**f**) IL-10 secretion was determined by flow cytometry and ELISA, respectively. **g** TRAPs were pretreated with Proteinase K for 2 h at 55 °C, DNase I for 1 h at 37 °C or RNase for 3 h at 37 °C, respectively, followed by incubation with BMDMs for 72 h. PD-L1 was evaluated by flow cytometry, and (**h**) IL-10 was tested by ELISA. Data are shown as mean ± SEM, and are representative of three independent experiments. ***p < 0.001 by unpaired *t* test. (**d**, **f** and **h**)

We further clarified TRAPs-induced signals in macrophage polarization, and focused on the p38 pathway, which was reported to have a pivotal role in the induction of PD-L1 and IL-10 [36] and IL-4-induced alternative activation of macrophages [37]. After stimulation of BMDMs with TRAPs, p38 phosphorylation was observed at 0.5 h, and declined rapidly after 1 h to the control level at 4 h. Phosphorylation of STAT3 was detectable at 2 h, and increased thereafter (Fig. 4a). Next, we determined whether p38 was required for TRAPs-induced STAT3 activation. Consistent with this hypothesis, inhibition of p38 activation by the specific inhibitor SB203580 repressed STAT3 phosphorylation (Fig. 4b) and the induction of PD-L1 and IL-10 (Fig. 4c and d). Pretreatment of macrophages with the STAT3 inhibitor Stattic also significantly diminished PD-L1 and IL-10 induction (Fig. 4e and f). Treatment of TRAPs with proteinase K, but not DNase or RNase, significantly blocked the upregulation of PD-L1 and IL-10 (Fig. 4g and h). Taken together, these findings indicate that macrophages polarization is induced by protein components on TRAPs via activation of the p38-STAT3 pathway.

### Inhibition of autophagy in tumor cells retards tumor growth and enhances antitumor responses

**Fig. 5:**
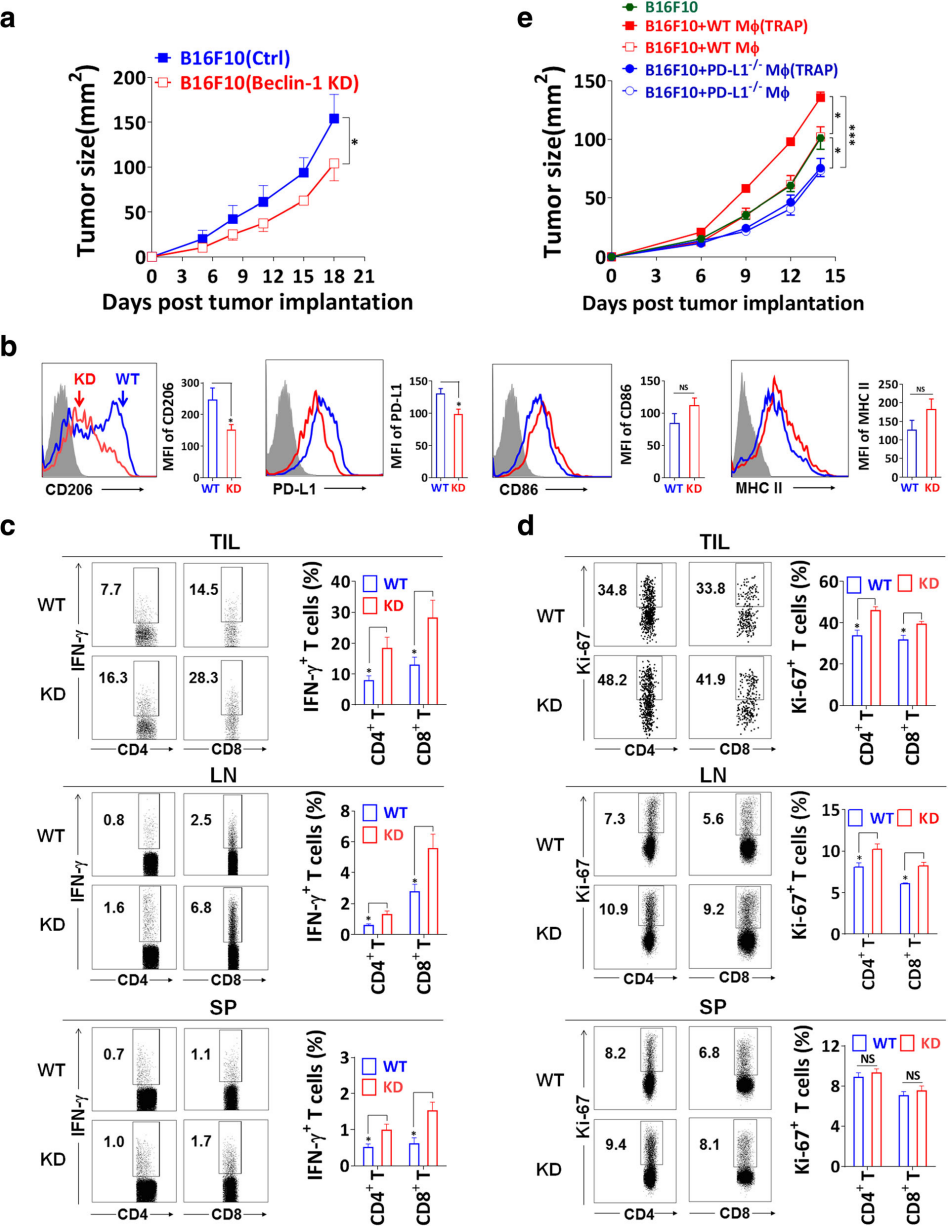
Knockdown of Beclin1 inhibits tumor growth and alters TAMs polarization. **a** BECN1-KD B16F10 and Ctrl-B16F10 cells were s.c. implanted to C57BL/6 mice (*n* = 6 per group). Tumor area was measured at the described days. **b** Tumor bearing mice were euthanized around day 15, total TAMs (F4/80^+^ CD11b^+^) were gated and analyzed for mean fluorescent intensity (MFI) of CD206, PD-L1, CD86 and MHC II expression by flow cytometry. Events are gated on singlet, live and CD45^+^ tumor infiltrating cells. **c** TILs were stimulated with Cell Stimulation Cocktail for 5 h, the frequency of CD4^+^ IFN-γ^+^ and CD8^+^ IFN-γ^+^ T cells was determined by intracellular staining. Single suspension cells from dLNs and spleens were stimulated with mitomycin C inactivated B16F10 cells at a ratio of 30:1 for 21 h, breferdin A plus monensin was added for the last 5 h of culture, and IFN-γ production was determined by flow cytometry. **d** T cells from TILs, dLNs and spleens were tested for Ki-67 expression by intracellular staining. **e** B16F10 cells were mixed with WT or PD-L1^−/−^ BMDMs (2:1) treated with or without TRAPs and then injected s.c. to C57BL/6 mice (n = 6 per group). Tumor growth was monitored at the indicated days. Data (mean ± SEM) are representative of three independent experiments. *p < 0.05 and ***p < 0.001 by unpaired *t* test (**a**-**d**) and two-way ANOVA (**e**)

To determine whether TRAPs favored the tumor-promoting characteristics of TAMs, we established B16F10 cells that stably express shRNA targeting the central autophagy regulator Beclin1 [22] (Additional file 2: Figure S4a). Beclin1 knockdown caused lower intracellular LC3-II accumulation, blocked the enhancement of LC3-II induced by chloroquine (Additional file 2: Figure S4b), and reduced TRAPs secretion (Additional file 2: Figure S4c) in Beclin1 knockdown B16F10 cells. When inoculated into C57BL/6 mice, Beclin1 knockdown cells exhibited a significant delay of growth (Fig. 5a). Of note, stable inhibition of autophagy in tumor cells in vivo was confirmed over the duration of the study, as evidenced by the reduction of Beclin1 and LC3-II in cell lysates of tumors harvested around 20 days after implantation (Additional file 2: Figure S4d). Meanwhile, TRAPs from BECN1-KD B16F10 cells had reduced ability to induce PD-L1 and IL-10 expression on BMDMs compared with TRAPs from Ctrl-B16F10 cells (Additional file 2: Figure S4e and f).

We postulated that the low tumor burden caused by Beclin1 silencing may be related to altered tumor immune microenvironment, including the functional properties of TAMs. TAMs from mice bearing Beclin1 knockdown tumors had significantly decreased expression of CD206 and PD-L1, as well as slightly increased expression of CD86 and MHC-II whereas the effect did not achieve statistical significance (Fig. 5b), indicating that loss of tumor cell autophagy led to TAMs reprogramming from an immunosuppressive to an inflammatory phenotype. Furthermore, a higher frequency of IFN-γ-producing T cells was observed in Beclin1 knockdown tumors. Re-stimulation of T cells from spleens and dLNs also revealed a higher proportion of IFN-γ^+^ CD4^+^ and CD8^+^ T cells in mice bearing Beclin1 knockdown tumors (Fig. 5c). Intratumoral and dLNs T cells in mice bearing Beclin1 tumors expressed higher level of the proliferation marker Ki-67 (Fig. 5d).

To further determine whether PD-L1 upregulation by TRAPs-treated BMDMs could facilitate tumor growth, B16F10 cells were co-injected with WT or PD-L1-deficient (*Pdcd1l1*^−/−^) BMDMs treated with or without TRAPs, respectively. Mice co-injected with *Pdcd1l1*^−/−^ BMDMs experienced slower tumor growth than WT BMDMs with or without TRAPs pretreatment. Co-injection of TRAPs-stimulated WT BMDMs significantly accelerated tumor growth compared to the co-injection of control WT BMDMs. Most importantly, TRAPs treatment of WT but not *Pdcd1l1*^−/−^ BMDMs resulted in larger tumors as compared to the untreated group (Fig. 5e). These results corroborate the conclusion that TRAPs-educated macrophages rely on PD-L1 induction to dampen T-cell mediated antitumor immune responses to foster tumor progression.

### Autophagosomes from cancer patients induce monocyte polarization to M2-like phenotype with immunosuppressive activities

**Fig. 6:**
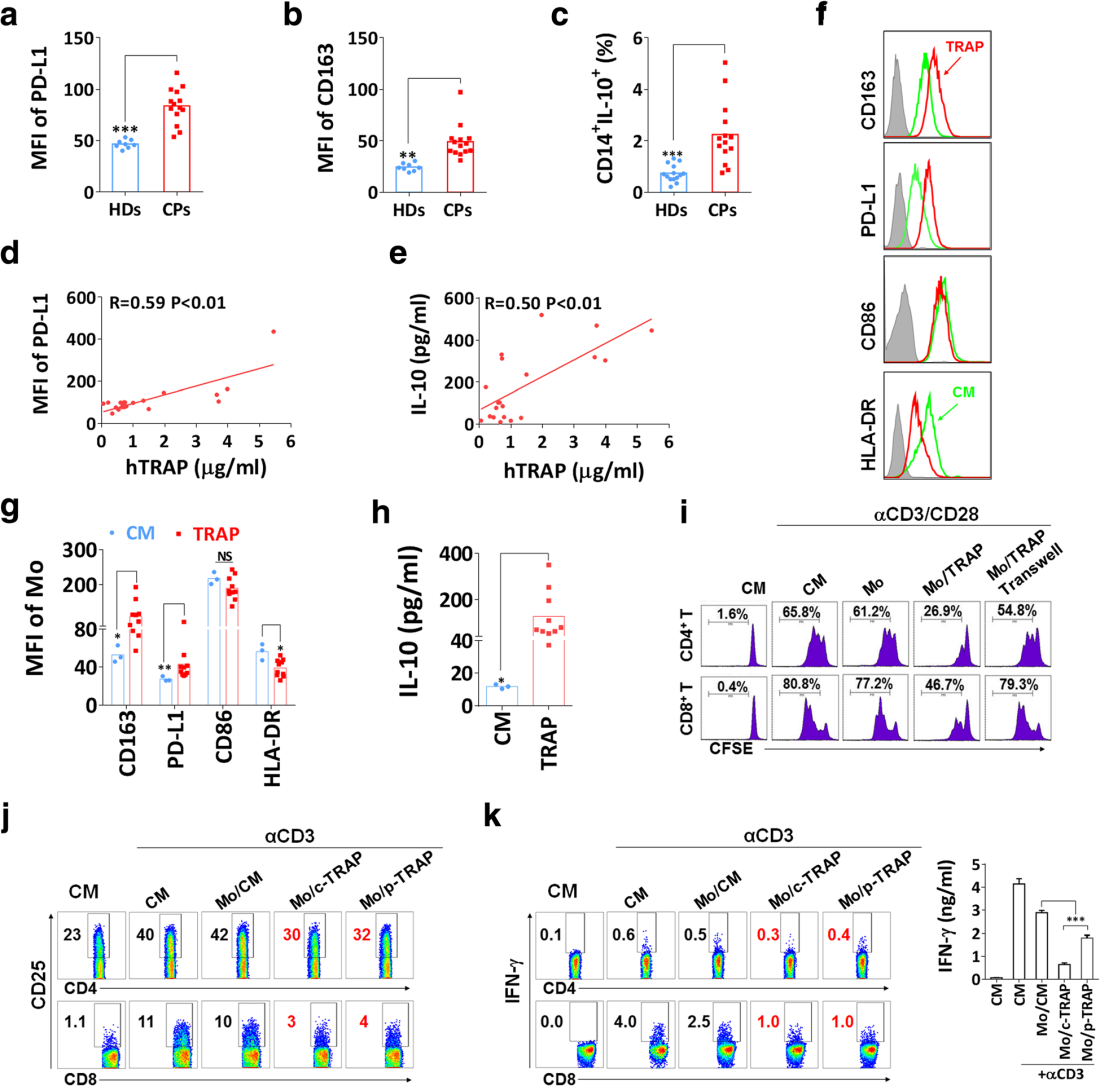
TRAPs derived from malignant pleural effusions or ascites of cancer patients regulate monocytes polarization. **a**, **b** The MFIs of (**a)** CD163 and (**b**) PD-L1 on CD14^+^ monocytes from peripheral blood of cancer patients (CPs) (*n* = 14) and healthy donors (HDs) (*n* = 8) were assessed by flow cytometry. **c** PBMCs from CPs and HDs were stimulated with LPS (100 ng/ml) for 18 h, breferdin A plus monensin was added for the last 6 h of culture, and the frequency of CD14^+^ IL-10^+^ was determined by flow cytometry. **d**, **e** The graphs showed the correlation between concentration of TRAP and MFI of PD-L1 on CD14^+^ monocytes and total IL-10 in malignant pleural effusions or ascites of cancer patients (*n* = 20). **f-h** Purified CD14^+^ monocytes from healthy donors were treated with TRAPs (5 μg/ml) derived from pleural effusions or ascites of cancer patients (*n* = 10) for 3 d. **f**, **g** The expression of CD163, PD-L1, CD86 and HLA-DR was detected by flow cytometry. **h** The amount of IL-10 in the supernatant was assessed by ELISA. **i** Analysis of T cell proliferation by flow cytometry. Monocytes were left untreated or pretreated with TRAPs for 3 d, then incubated with CFSE-labeled autologous T cells (1:3) for 5 d in the presence of anti-CD3 and anti-CD28, or monocytes and T cells were separated by a transwell chamber. **j**, **k** Monocytes were left untreated or pretreated with TRAPs from MDA-MB-231 cells (c-TRAP) and cancer patient (p-TRAP) for 3 d, then incubated with autologous T cells (1:3) for 20 h in the presence of immobilized anti-CD3. **j** CD25 expression on T cells was examined by flow cytometry. **k** Breferdin A plus monensin was added during the last 8 h of culture. The percentage of CD4^+^ IFN-γ^+^ and CD8^+^ IFN-γ^+^ T cells and total IFN-γ secretion in the supernatant was detected by flow cytometry and ELISA, respectively. Data (mean ± SEM) are representative of three independent experiments. *p < 0.05, **p < 0.01 and ***p < 0.001 by unpaired *t* test. (**a**, **b**, **c**, **g**, **h** and **k**) and Spearman’s rank correlation (**d** and **e**)

To further determine whether autophagosomes from cancer patients contribute to the formation of immunosuppressive TAMs, 25 malignant pleural effusions or ascites specimens were collected, and their clinical characteristics were depicted in (Additional file 1: Table S1). The samples contain abundant vesicles that were similar in size, morphology and the expression of typical markers to the previously described autophagosomes isolated from cell culture supernatant (Additional file 2: Figure S1d–f). Elevation of CD163, PD-L1 and IL-10 expression on CD14^+^ monocytes from PBMCs of cancer patients was observed as compared to those from healthy donors (Fig. 6a–c). Moreover, the results showed a significant positive correlation between the concentration of LC3B^+^ autophagosomes and the expression of PD-L1 and IL-10 in matched monocytes from effusions or ascites of cancer patients (Fig. 6d and e). Peripheral blood CD14^+^ monocytes from healthy donors treated with TRAPs from cancer patients exhibited a significant increase in CD163 and PD-L1 expression and a decrease in HLA-DR expression. TRAPs stimulation showed the trend of downregulating CD86 expression, although the effect did not achieve statistical significance (Fig. 6f and g). Meanwhile, monocytes produced high level of IL-10 following TRAPs stimulation (Fig. 6h). Intriguingly, we also found that LC3B^+^ EVs, isolated from malignant ascites of a lung cancer patient (Additional file 2: Figure S6a), were more potent than LC3B^−^ EVs to upregulate CD163, PD-L1 and IL-10 expression and downregulate HLA-DR expression of monocytes (Additional file 2: Figure S6b-d), suggesting that LC3B^+^ EVs (TRAPs) are a dominant subtype of large EVs in converting monocytes.

To verify the immunoregulatory effects of TRAPs-treated monocytes, we cultured these cells with CFSE-labeled autologous T cells in the presence of anti-CD3 and anti-CD28. Consistent with our observations in mouse BMDMs, TRAPs-activated monocytes acquired the ability to suppress the proliferation of both CD4^+^ and CD8^+^ T cells (Fig. 6i). Meanwhile, TRAPs-pretreated monocytes suppressed the expression of CD25 on T cells (Fig. 6j), and diminished the frequency of IFN-γ^+^ T cells and IFN-γ secretion into the supernatant (Fig. 6k). Collectively, autophagosomes from malignant pleural effusions or ascites of cancer patients were able to modulate TAMs polarization toward an M2-like phenotype with potent immunosuppressive functions.

## Discussion

**Fig. 7:**
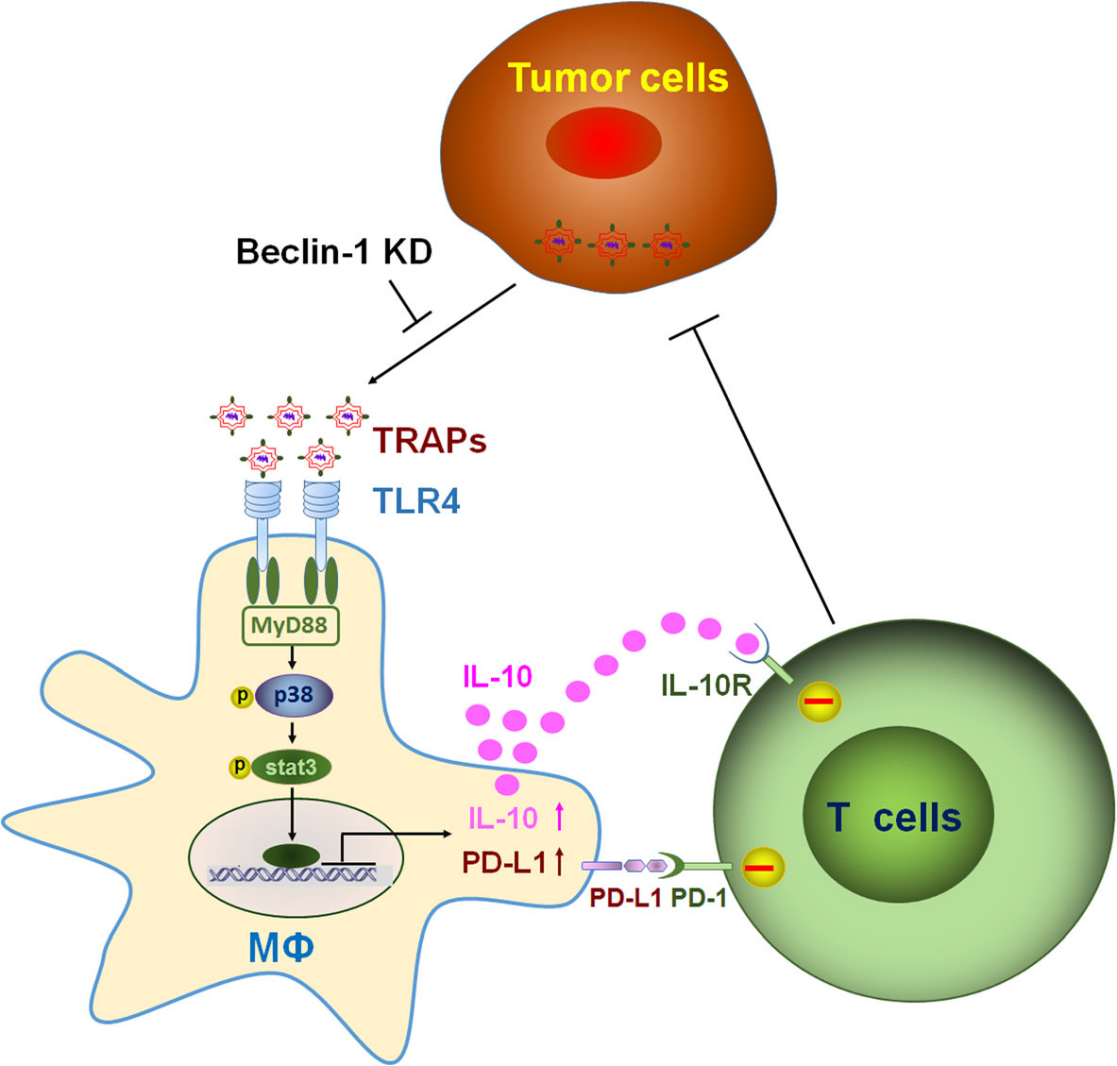
Proposed schematic model depicting the role of TRAPs in macrophage polarization and adaptive immune suppression. TRAPs are uptaken by macrophages and promotes MyD88-p38-STAT3 activation via TLR4, leading to upregulation of PD-L1 and IL-10 that is responsible for inhibition of cytotoxic T lymphocytes (CTLs) activation, and in turn enhancing tumor progression

TAMs can be polarized into tumor-promoting phenotype with immune modulatory effects by tumor-derived instructive signals within the TME, such as CSF-1 [38], lactate [39] and PGE2 [40]. Recently, extracellular vesicles have also been shown to be associated with TAMs polarization [41, 42]. In the present study, we observed that (i) TRAPs from murine tumor cell lines as well as malignant pleural effusions and ascites of cancer patients were internalized by macrophages and skewed macrophages to an M2-like phenotype with increased expression of PD-L1 and IL-10; (ii) blocking PD-L1 on macrophages significantly abrogated TRAPs-mediated inhibition of T cell proliferation and promotion of tumor growth; (iii) TLR4-MyD88-p38-STAT3 signaling pathway was instrumental for TRAPs-induced upregulation of PD-L1 and IL-10 expression in macrophages; (iv) interference with autophagosome formation in tumor cells inhibited tumor growth and blocked M2-like macrophage polarization. Our results revealed that TRAPs are a novel type of EVs capable of conferring pro-tumor properties to TAMs (Fig. 7).

During classical autophagy, autophagosomes fuse with lysosomes to degrade cytoplasmic cargo. However, we have shown an alternative destiny of the autophagosomes, which is tumor cells release them to the extracellular milieu through currently unknown mechanisms [33]. Importantly, autophagosomes can serve as vehicles to transfer a broad spectrum of contents, including tumor antigens and DAMPs (HSPs, HMGB1, S100 proteins, DNA and RNA) to different types of target cells. Inhibition of the degradative function of proteasome and lysosome, a wider repertoire of tumor-associated antigens and DAMPs were enriched in autophagosomes. These artificial autophagosomes, termed DRibble (DRiPs-containing blebs), could be utilized as a potent cancer vaccine to efficiently activate innate and adaptive immune responses [33, 43]. Nevertheless, our observations also suggest that naturally released autophagosomes, called TRAPs, exert immune suppressive roles by regulating the functions of B cells and neutrophils [31, 32]. Although recent literatures emphasized the importance of exosomes derived from chronic lymphocytic leukemia (CLL) and glioblastoma in eliciting tumor-supportive TAMs [41, 42, 44], TRAPs described here are vesicles that are biochemically and functionally distinct from exosomes. TRAPs are 300–900 nm sized double-membrane structure vesicles that express the typical autophagosome marker LC3-II but not the exosome marker CD63, and could be pelleted at a low centrifugation speed (12,000 g). We also excluded the interference of other extracellular vesicles, by purifying TRAPs with LC3B magnetic beads.

Treatment of BMDMs with TRAPs induced mixed M2-like macrophage polarization. In addition to IL-10, TRAPs-induced BMDMs also produced significant levels of proinflammatory cytokines, including IL-1β and IL-6, but no IL-12. In fact, these two proinflammatory cytokines were reported to be highly proangiogenic in the TME and essential for tumor progression [45]. Intriguingly, we observed the upregulation of PD-L1 on macrophages after treatment with TRAPs. Although tumor cells were initially regarded as the predominant source of PD-L1 for immune suppression, clinical trials showed that cancer patients with PD-L1-negative tumors can benefit from PD-1/PD-L1 blockade therapy, implying the involvement of PD-L1 on host cells in this effect [46]. Recently, emerging data indicated that PD-L1 on myeloid cells, including DCs and macrophages, but not on tumor cells, determined the response to PD-L1 blockade in mice [47, 48]. Furthermore, the percentage of PD-L1^+^ macrophages correlated with the clinical efficacy of PD-1/PD-L1 blockade in melanoma and ovarian cancer. We found TRAPs-stimulated BMDMs could inhibit T-cell proliferation in response to the nonspecific anti-CD3/CD28 stimulus and the specific OVA_257–264_ stimulus. PD-L1 had a major role in suppression, and the simultaneous blockade of PD-L1 and IL-10 almost completely restored T cell proliferation.

A growing body of research suggested that endogenous TLRs ligands not only have immune stimulatory effects but also engender immune tolerance via the induction of several immunosuppressive elements, such as Tregs, PD-L1 and IL-10 [49]. Our data showed that TLR4 was a key receptor for TRAPs-mediated macrophage signaling. TLR2 ablation only diminished the production of IL-10, whereas TLR4 ablation led to the reduction of both IL-10 and PD-L1. Importantly, the induction of IL-10 and PD-L1 was almost completely abrogated in *Myd88*^−/−^ BMDMs. In line with this, MyD88 deficiency completely abolished TRAPs-treated BMDMs mediated inhibition of T cell proliferation, whereas ablation of TLR4 only partially restored T cell proliferation. MAPKs have been considered as a major pathway involved in TLR signaling. We found that PD-L1 and IL-10 expression was partly attenuated after blocking p38. Conversely, the inhibition of Erk1/2 or JNK had no effect on the expression of PD-L1 (Additional file 2: Figure S5a). STAT3, a transcription regulator of M2 related molecules [50], appeared critical in our system, as blocking of TRAPs-induced STAT3 activation impaired the upregulation of IL-10 and PD-L1 on BMDMs. However, there appeared no association between autocrine stimuli and PD-L1 expression in our experimental settings, as blocking IL-6 or IL-10, failed to decrease TRAPs-induced PD-L1 expression (Additional file 2: Figure S5a), suggesting that additional factors or cytokine-independent mechanisms were involved in the modulation of PD-L1. Consistent with our observations, mounting evidences have identified a decisive role for TLR4 signaling in direct STAT3 activation and subsequent PD-L1 expression [50]. Future studies will explore whether other receptors or signaling pathways are involved. An important question is which components on TRAPs trigger TLRs and the downstream signaling. Pretreatment of TRAPs with proteinase K, but not DNase or RNase, destroyed the stimulatory potential of TRAPs, suggesting that the triggering ligands are proteins. We previously showed that HMGB1 on TRAPs was involved in inducing IL-10^+^ Bregs [31]. However, HMGB1 had no effect on the induction of M2-like macrophages. Further studies are needed to identify the determinants responsible for macrophage polarization.

Although conflicting findings have been observed concerning the role of autophagy in advanced cancer, autophagy has been identified as a promising target in cancer therapy. Autophagy inhibition not only impacts tumor cells directly, but also may have antitumor effects by affecting other constituents of the TME [51–53]. Indeed, we observed that TRAPs secreted by Beclin1-silenced B16F10 cells contained less LC3-II and this correlated with reduced ability to induce PD-L1 and IL-10. Intriguingly, TAMs isolated from Beclin1 knockdown tumors displayed a less immunosuppressive phenotype with decreased levels of CD206 and PD-L1. Furthermore, Beclin1 deficiency in tumor cells boosted the functions of T cells as reflected by a higher production of IFN-γ and higher percentage of Ki67^+^ TILs. This finding supports the model in which Beclin1-dependent formation of TRAPs in B16F10 cells has a crucial role in TAMs polarization. Conversely, melanomas co-implanted with TRAPs-treated BMDMs had significant tumor progression compared to tumors co-injected with untreated BMDMs. Importantly, the tumor-promoting function of TRAPs-treated BMDMs in vivo was mainly dependent on PD-L1.

Besides tumor cells, many other cell types were also found in effusions and ascites, such as monocytes, DCs, T cells [54]. Although we did not ascertain whether autophagosomes are secreted exclusively by cancerous cells, we demonstrated that LC3-II^+^ autophagosomes from malignant pleural effusions or ascites of cancer patients were capable of transforming human monocytes into those that significantly suppress the proliferation and effector functions of T cells. More importantly, we found TRAP was a dominant subtype of large EVs in converting monocytes to a M2-like phenotype, as reflected by elevated expression of CD163, PD-L1, IL-10 and decreased expression of HLA-DR on monocytes when compared with large LC3B^−^ EVs. In addition, PD-L1 and IL-10 expression in matched monocytes, which were collected from effusions or ascites of cancer patients, was correlated with the levels of LC3B^+^ EVs in the same cohort. Our studies provide new insight for the role of TRAPs, a key subpopulation of large EVs, in the tumor microenvironment that promotes the development of PD-L1-high TAMs, and highlight that TRAPs could be an important therapeutic target to reverse the immunosuppressive tumor microenvironment. Future studies will focus on exploring the correlation between TRAP levels in peripheral blood or ascites and the pathological characteristics and disease progression of clinical cancer patients.

## Conclusions

In this study, we demonstrate that TRAPs are a novel mechanism exploited by tumor cells for immune suppression, including inducing M2 polarization as reflected by increased expression of PD-L1 and IL-10. This effect is mainly dependent on TLR4-mediated MyD88-p38-STAT3 signaling pathway. Blocking autophagy in tumor cells promoted the switch of TAMs from immunosuppressive M2-like to the antitumor M1-like phenotype and restored immune function of TILs. These observations offer a strong rationale for targeting autophagy as a therapeutic approach to improve the efficacy of anti-PD-1 or anti-PD-L1-based cancer immunotherapy.

## Additional files

Additional file 1: **Table S1.** Clinical characteristics of 25 patients presenting with malignant pleural effusions or ascites. **Table S2.** Primers used in Real-time quantitative PCR analyses. (PDF 536 kb)

Additional file 2: **Figure S1.** Characterization of TRAPs from tumor cell lines or cancer patients. **Figure S2.** Phenotype determination of BMDMs stimulated by TRAPs with different doses and origin. **Figure S3.** TRAPs treated BMDMs inhibit T cell proliferation. **Figure S4.** Genetic inhibition of autophagy by targeting Beclin1 reduces TRAPs production. **Figure S5.** TRAPs induced PD-L1 upregulation on BMDMs was mainly dependent on p38 activation. **Figure S6.** Comparison of LC3B^+^ EVs and LC3B^-^ EVs in converting monocytes. (DOCX 1421 kb)

## Abbreviations

APCs: Antigen-presenting cells
Arg1: Arginase 1
BMDMs: Bone marrow derived macrophages
CFSE: Carboxyfluorescein succinimidyl ester
CLL: Chronic lymphocytic leukemia
CPRG: Chlorophenol red-β-D-galactopyranoside
CTLs: Cytotoxic T lymphocytes
DAMPs: Damage-associated molecular pattern molecules
DCs: Dendritic cells
dLNs: Draining lymph nodes
Dribble: DRiPs-containing blebs
EVs: Extracellular vesicles
HSP27: Heat shock protein 27
KD: Knock down
KO: Knock out
mAb: Monoclonal antibody
NOS2: Nitric oxide synthase 2
PBMC: Peripheral blood mononuclear cell
ROS: Reactive oxygen species
shRNA: Short hairpin RNA
TAMs: Tumor-associated macrophages
TEM: Transmission Electron Microscopy
TLRs: Toll-like receptors
TME: Tumor microenvironment
TRAPs: Tumor cell-released autophagosomes
Treg: CD4^+^ regulatory T cells
WT: Wild-type
