# Supplementary material for: Tumor cell-released autophagosomes (TRAPs) promote immunosuppression through induction of M2-like macrophages with increased expression of PD-L1
Source: J Immunother Cancer. 2018 Dec 18;6:151. doi: 10.1186/s40425-018-0452-5 (PMC6299637; doi:10.1186/s40425-018-0452-5)
Supplement: Supplementary file 1 — Table S1. Clinical characteristics of 25 patients presenting with malignant pleural effusions or ascites. Table S2. Primers used in Real-time quantitative PCR analyses. (PDF 536 kb) [file 40425_2018_452_MOESM1_ESM.pdf]

Additional file 1

Additional file 1: Table S1

Table S1. Clinical characteristics of 25 patients presenting with malignant pleural effusions or ascites

Patient No.	Age/Sex	Primary Tumor site	Histologic Diagnosis	Pleural effusions /ascites	Stage
01	54/F	Ovary	Adenocarcinoma	Ascites	Stage III
02	61/F	Ovary	Adenocarcinoma	Ascites	Stage IV
03	47/F	Ovary	Adenocarcinoma	Ascites	Stage III
04	53/F	Ovary	Adenocarcinoma	Ascites	Stage IV
05	58/F	Ovary	Adenocarcinoma	Ascites	Stage IV
06	65/F	Ovary	Adenocarcinoma	Ascites	Stage IV
07	55/F	Ovary	Adenocarcinoma	Ascites	Stage IV
08	51/F	Lung	Adenocarcinoma	Pleural effusions	Stage IV
09	67/F	Lung	Adenocarcinoma	Pleural effusions	Stage IV
10	75/F	Lung	Adenocarcinoma	Pleural effusions	Stage IV
11	72/M	Lung	Adenocarcinoma	Pleural effusions	Stage III
12	68/M	Lung	Adenocarcinoma	Pleural effusions	Stage IV
13	74/M	Lung	Adenocarcinoma	Pleural effusions	Stage IV
14	77/M	Lung	Adenocarcinoma	Ascites	Stage IV
15	54/M	Lung	Adenocarcinoma	Pleural effusions	Stage IV
16	69/M	Lung	Adenocarcinoma	Pleural effusions	Stage IV
17	57/M	Stomach	Adenocarcinoma	Pleural effusions	Stage IV
18	63/M	Stomach	Adenocarcinoma	Pleural effusions	Stage IV
19	74/M	Stomach	Adenocarcinoma	Ascites	Stage IV
20	70/M	Cardia	Adenocarcinoma	Pleural effusions	Stage IV
21	75/F	Cardia	Adenocarcinoma	Pleural effusions	Stage IV
22	55/F	Breast	Adenocarcinoma	Pleural effusions	Stage IV
23	64/M	Colon	Colorectal adenocarcinoma	Ascites	Stage IV
24	49/F	Liver	Hepatocellular carcinoma	Ascites	Stage IV
25	65/M	Pancreas	Adenocarcinoma	Ascites	Stage IV

Key: M: Male; F: Female

Additional file 1: Table S2

Table S2. Primers used in Real-time quantitative PCR analyses

**Target Gene**		**Sequence (5' → 3')**
GAPDH	Forward	TGAAGCAGGCATCTGAGGG
	Reverse	CGAAGGTGGAAGAGTGGGAG
NOS2	Forward	CCAAGCCCTCACCTACTTCC
	Reverse	CTCTGAGGGCTGACACAAGG
IL-10	Forward	GCTCTTACTGACTGGCATGAG
	Reverse	CGCAGCTCTAGGAGCATGTG
PD-L1	Forward	AAAGTCAATGCCCCATACCG
	Reverse	TTCTCTTCCCACTCACGGGT
Arginase1	Forward	ACCTGGCCTTTGTTGATGTCCCTA
	Reverse	AGAGATGCTTCCAACTGCCAGACT
Beclin1	Forward	ACTGGGTTTTGATGGAATAGG
	Reverse	TAAGGAGTTGCCGTTATACTGT
