# Supplementary material for: Tumor cell-released autophagosomes (TRAPs) promote immunosuppression through induction of M2-like macrophages with increased expression of PD-L1
Source: J Immunother Cancer. 2018 Dec 18;6:151. doi: 10.1186/s40425-018-0452-5 (PMC6299637; doi:10.1186/s40425-018-0452-5)
Supplement: Supplementary file 2 — Figure S1. Characterization of TRAPs from tumor cell lines or cancer patients. Figure S2. Phenotype determination of BMDMs stimulated by TRAPs with different doses and origin. Figure S3. TRAPs treated BMDMs inhibit T cell proliferation. Figure S4. Genetic inhibition of autophagy by targeting Beclin1 reduces TRAPs production. Figure S5. TRAPs induced PD-L1 upregulation on BMDMs was mainly dependent on p38 activation. Figure S6. Comparison of LC3B+ EVs and LC3B- EVs in converting monocytes. (DOCX 1421 kb) [file 40425_2018_452_MOESM2_ESM.docx]

**Additional file 2**


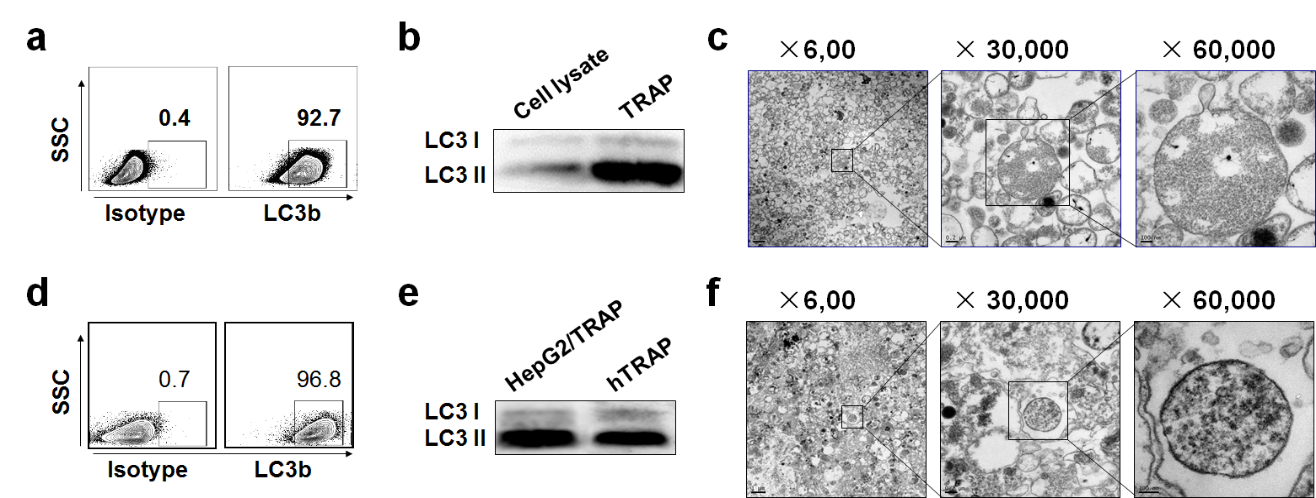


**Figure S1.** Characterization of TRAPs from tumor cell lines or cancer patients. **a-c** Characterization of B16F10 TRAPs. **a** Flow cytometry analysis of LC3B on TRAPs. **b** Western blot analysis of LC3-II for parental cells and TRAPs. **c** TEM images of TRAPs. Scale bar, 1 μm, 0.2 μm, 100 nm (from left to right). **d-f** Characterization of TRAPs derived from cancer patients. **d** Flow cytometry analysis of LC3B on TRAPs. **e** Western blot analysis of LC3-II for TRAPs from HepG2 cells and cancer patients. **f** TEM images of TRAPs. Scale bar, 1 μm, 0.2 μm, 100 nm (from left to right). Results are representative of three independent experiments.


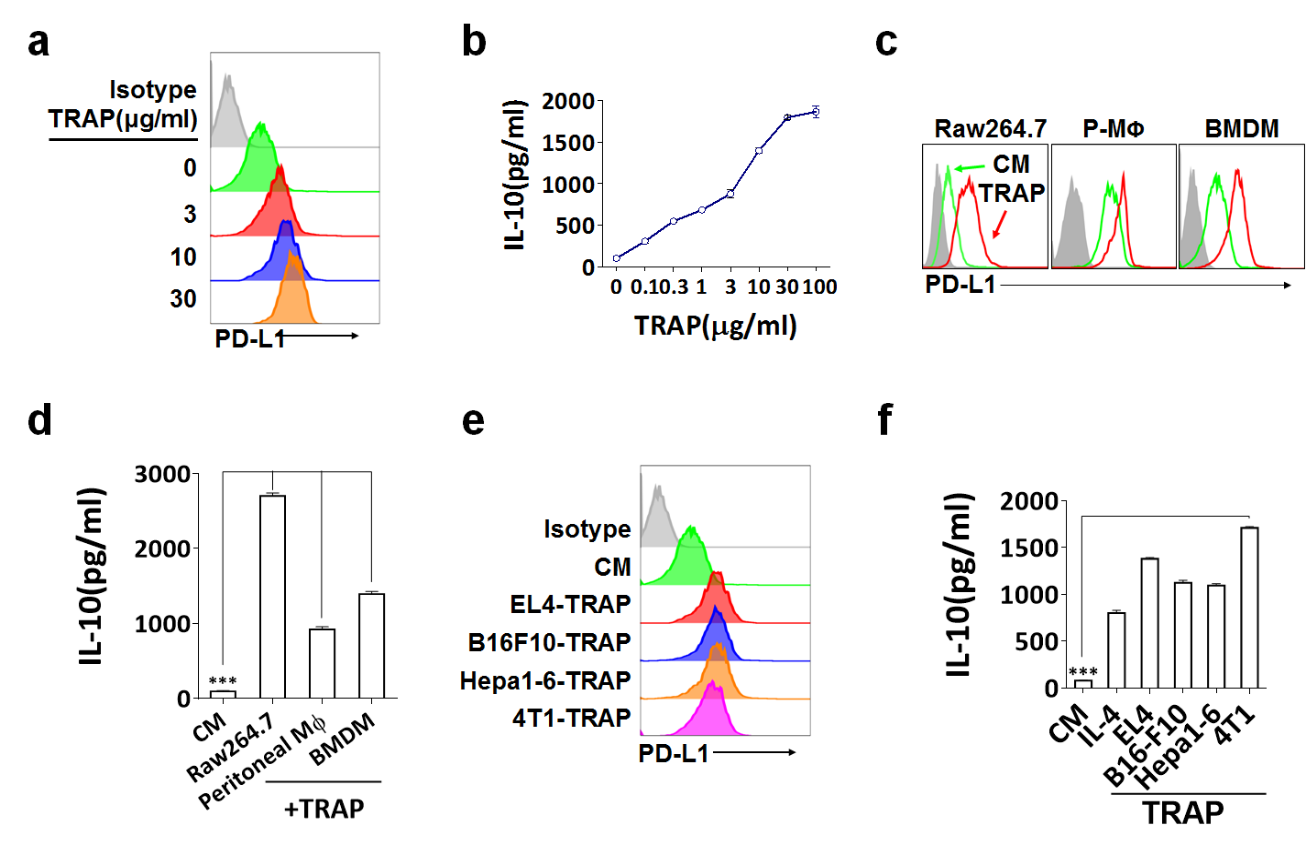


**Figure S2.** Phenotype determination of BMDMs stimulated by TRAPs with different doses and origin. **a** BMDMs were stimulated with TRAPs at indicated doses for 48 h. PD-L1 was evaluated by flow cytometry, and (**b**) IL-10 was measured by ELISA. **c** TRAPs were cultured with Raw264.7, peritoneal macrophage and BMDMs, respectively. PD-L1 and (**d**) IL-10 was detected after 48 h. **e** BMDMs were treated with TRAPs from EL4, B16F10, Hepa1-6 or 4T1 cells for 48 h, followed by assessment of PD-L1 and (**f**) IL-10. Data (mean ± SEM) are representative of three independent experiments. ****p* < 0.001 by unpaired *t* test (d and f).


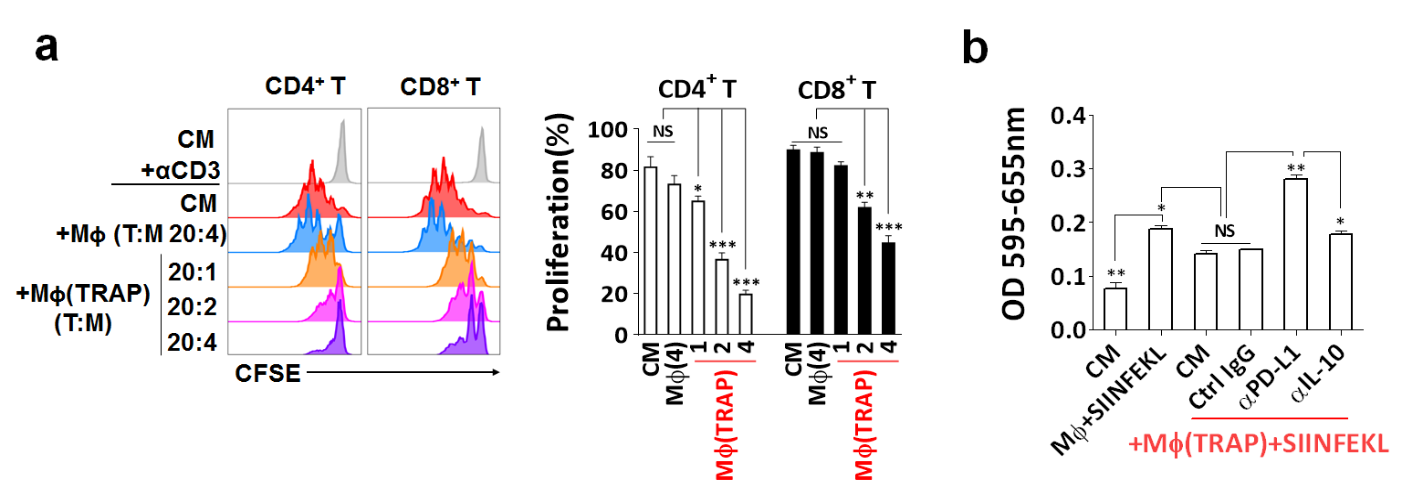


**Figure S3.** TRAPs treated BMDMs inhibit T cell proliferation. **a** CFSE-labeled T cells were left untreated or were activated in the presence of plate-bound anti-CD3 plus soluble anti-CD28 mAb and were either cultured alone or were incubated with control or TRAPs (10 μg/ml) stimulated BMDMs at indicated ratios (20:1, 20:2 and 20:4). Cells were harvested after 72 h, and T cells division was analyzed by flow cytometry. **b** BMDMs were loaded with peptide SIINFEKL (1 μg/ml) for 2 h, washed, and were then cocultured with B3Z cells for 18 h at a ratio of 1:3, in the presence of anti-PD-L1 (10 μg/ml), anti-IL-10 (10 μg/ml), or IgG isotype control (10 μg/ml). B3Z T cell activation was measured by CPRG assay. Data are representative of three independent experiments. **p* < 0.05, ***p* < 0.01 and ****p* < 0.001 by unpaired *t* test (a and b).

**
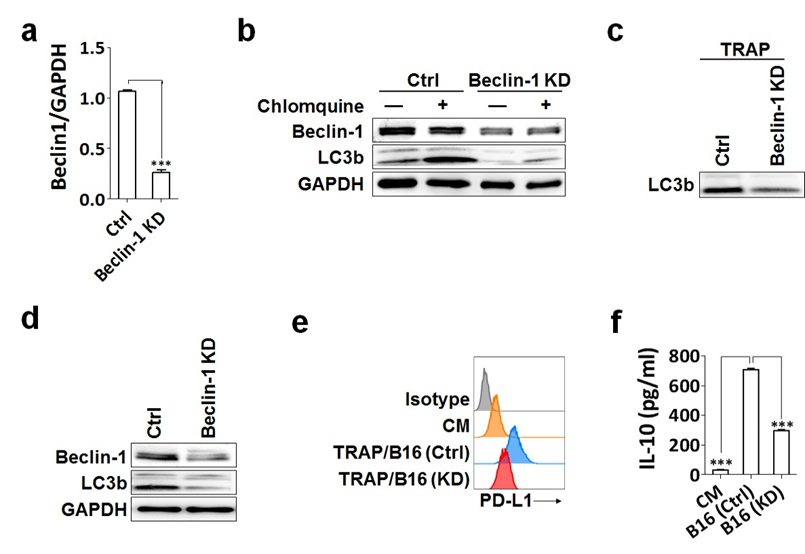
**

**Figure S4.** Genetic inhibition of autophagy by targeting Beclin1 reduces TRAPs production. **a** qRT-PCR analysis of *Beclin1* mRNA expression in B16F10 (Ctrl) and B16F10 (BECN1 KD) cells. **b** Tumor cells were treated with or without chlomquine (30 μM) for 24 h, lysates were detected for Beclin1 and LC3B expression by western blot. **c** TRAPs were obtained from equal number of B16F10 (Ctrl) and B16F10 (BECN1 KD) cells, suspended in same volume of PBS, then were detected for LC3-II by western blot. **d** Lysates from resected tumors were assessed for Beclin1 and LC3B by western blot (n = 6 per group). **e, f** BMDMs were treated with TRAPs from equal number of BECN1-KD B16F10 (B16 KD) and Ctrl-B16F10 (B16 Ctrl) cells for 24 h, expression of PD-L1 and IL-10 was detected by flow cytometry and ELISA, respectively. Data are representative of three independent experiments.


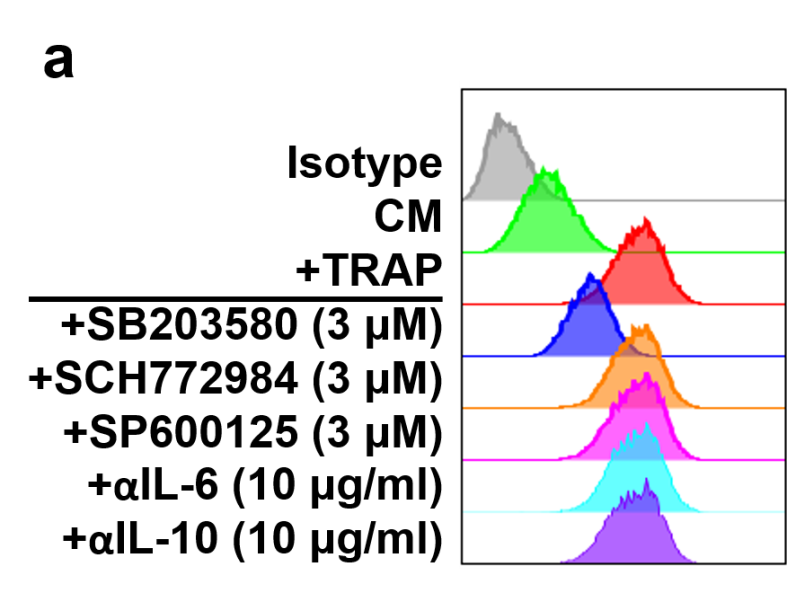


**Figure S5.** TRAPs induced PD-L1 upregulation on BMDMs was mainly dependent on p38 activation. **a** BMDMs were preincubated with p38 inhibitor SB203580 (3 μM), Erk1/2 inhibitor SCH772984 (3 μM), JNK inhibitor SP600125 (3 μM), anti-IL-6 mAb (10 μg/ml) and anti-IL-10 mAb (10 μg/ml) for 1 h, and then treated with TRAPs (10 μg/ml) for 48 h. Expression of PD-L1 was determined by flow cytometry.


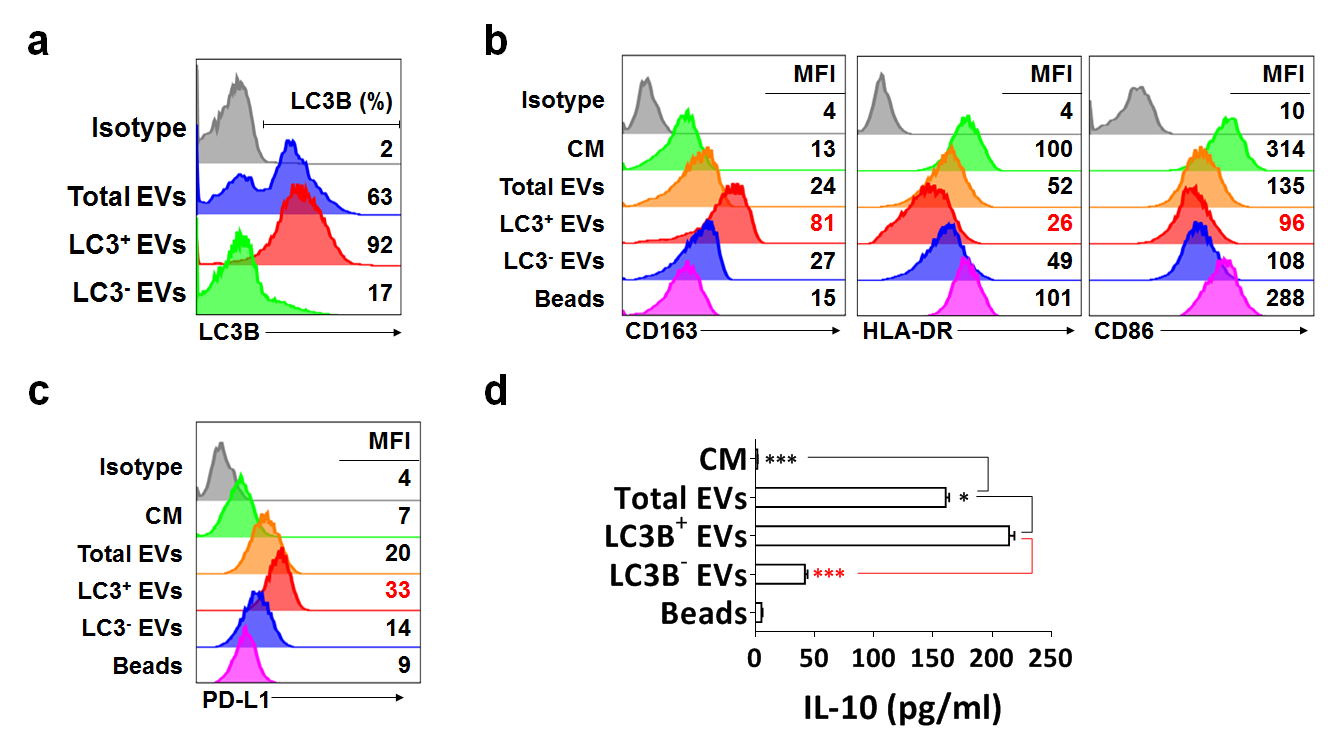


**Figure S6.** Comparison of LC3B^+^ EVs and LC3B^-^ EVs in converting monocytes. **a** LC3B^+^ EVs and LC3B^-^ EVs were sorted from total EVs of a lung cancer patient, and LC3B expression was determined by flow cytometry. **b-d** Purified CD14^+^ monocytes from healthy donors were treated for 3 d with total EVs, LC3B^+^ EVs, LC3B^-^ EVs (5 μg/ml) and magnetic beads, respectively. Expression of CD163, HLA-DR, CD86 (**b**) and PD-L1 (**c**) was detected by flow cytometry. IL-10 production (**d**) in the supernatant was assessed by ELISA.
